# The effects of clinical supervision on supervisees and patients in cognitive-behavioral therapy: a study protocol for a systematic review

**DOI:** 10.1186/s13643-017-0486-7

**Published:** 2017-05-11

**Authors:** Sven Alfonsson, Åsa Spännargård, Thomas Parling, Gerhard Andersson, Tobias Lundgren

**Affiliations:** 1 Centre for Psychiatry Research, Department of Clinical Neuroscience Karolinska Institutet and Stockholm Health Care Services, Norra Stationsgatan 69, SE-113 64 Stockholm, Sweden; 2 Department of Women’s and Children’s Health, Uppsala University, Uppsala, Sweden; 3 Department of Behavioural Sciences and Learning, Linköping University, Linköping, Sweden

**Keywords:** Systematic review, Clinical supervision, psychotherapy, Training

## Abstract

**Background:**

Clinical supervision by a senior therapist is a very common practice in psychotherapist training and psychiatric care settings. Though clinical supervision is advocated by most educational and governing institutions, the effects of clinical supervision on the supervisees’ competence, e.g., attitudes, behaviors, and skills, as well as on treatment outcomes and other patient variables are debated and largely unknown. Evidence-based practice is advocated in clinical settings but has not yet been fully implemented in educational or clinical training settings. The aim of this systematic review is to synthesize and present the empirical literature regarding effects of clinical supervision in cognitive-behavioral therapy.

**Methods:**

This study will include a systematic review of the literature to identify studies that have empirically investigated the effects of supervision on supervised psychotherapists and/or the supervisees’ patients. A comprehensive search strategy will be conducted to identify published controlled studies indexed in the MEDLINE, EMBASE, PsycINFO, and Cochrane Library databases. Data on supervision outcomes in both psychotherapists and their patients will be extracted, synthesized, and reported. Risk of bias and quality of the included studies will be assessed systematically.

**Discussion:**

This systematic review will rigorously follow established guidelines for systematic reviews in order to summarize and present the evidence base for clinical supervision in cognitive-behavioral therapy and may aid further research and discussion in this area.

**Systematic review registration:**

PROSPERO CRD42016046834

**Electronic supplementary material:**

The online version of this article (doi:10.1186/s13643-017-0486-7) contains supplementary material, which is available to authorized users.

## Background

Clinical supervision is an integral part of both psychotherapist training and continued professional development in many countries. Though there are variations, most clinical supervision takes place during basic psychotherapy training and many professional bodies and organizations endorse, promote, or regulate clinical supervision in standard psychiatric care as well. The definition of clinical supervision in psychotherapy varies to some degree but the fundamental aspect is a one-on-one tuition in which a supervisor helps a supervisee to develop theoretical knowledge, practical skills, and other kinds of therapeutic competence in regard to specific patient cases [1, 2]. Similar to other forms of professional development, such as taking courses and attending workshops, the ultimate goal of clinical supervision is that the psychotherapists provide safe and effective treatments for their patients [3]. Clinical supervision is thus justified based on this proposed causal chain between supervision, psychotherapist practice, and patient well-being [4]. Supervision has generally been viewed as a necessary and essential part of psychotherapist training but surprisingly, little empirical research has been conducted on the effects of clinical psychotherapy supervision, and the evidence for the causal mechanism in the educational pyramid is limited [5, 6]. In the lack of empirical guidelines, psychotherapy supervision has instead mostly been structured after models from psychotherapy practice.

There are several models of supervision emphasizing different aspects of supervision and supervisor behaviors but in a cognitive-behavioral therapy (CBT) context, supervision typically mimics the form and structure of psychotherapy [7, 8]. Supervision in CBT therefore includes features such as agenda setting, homework, problem solving, and feedback that can be derived from the therapy structure [9]. Clinical supervision in CBT was originally described by Padesky [10] and Liese and Beck [11] almost 20 years ago and has remained largely unchanged [12]. While this form of supervision may have high face validity in a CBT context, there is generally little empirical support for most of the specific supervision components that are recommended [13]. The relative low scientific standard and methodological deficits of many earlier studies on supervision effects makes it difficult to draw firm conclusions about even fundamental supervision components such as agenda setting and feedback [5, 13]. The methodological weaknesses of earlier studies include under developed theoretical framework, lack of validated measurements, and poor study designs and have been highlighted in previous reviews of clinical supervision [6, 14]. However, some specific supervision formats, such as video monitoring and feedback, may be effective in improving both psychotherapist competence and treatment outcomes [15]. In other words, the supervisions format may have a larger impact on supervisees than the supervision content but it is important to remember the generally low methodological standards of most studies and that direct empirical comparisons between different supervision models are rare or non-existent. In one of the first reviews on clinical supervision, Ellis, Ladany, Krengel, and Schult [16] found that the literature was dominated by qualitative or discursive papers that relatively few empirical studies had been conducted and that there were no key studies or seminal publications.

A few years later, in the only systematic review focusing specifically on CBT supervision, the main conclusion by Milne and James [17] was the lack of high-quality studies on psychotherapy supervision. Their systematic review did not rigorously follow standard review procedures and used a rather narrow inclusion criterion of only allowing behavioral outcome measures but the reasons for these methodological decisions are unknown. However, they did find some support for a positive effect of supervision on both supervisee competence and patient outcomes. They also identified positive effects from specific supervisory methods such as monitoring, providing feedback, modeling, and idiosyncratic instructions. Overall, the evidence base found by Milne and James was small and most of the reviewed research was conducted in the limited area of rehabilitation and disability education. It is therefore unclear whether the positive effects of supervision may be generalized to the broader context of psychotherapy supervision. On a positive note, Milne and James found examples of well-conducted research and remarked that empirical high-quality studies on supervision are clearly feasible.

In a later review on the effects of supervision, Wheeler and Richards [18] confirmed that psychotherapy supervision can have positive effects on supervisees, especially regarding self-awareness and therapist skills. However, the support for effects on patients was found to be rather weak and this conclusion has since been replicated in more recent studies [19]. Wheeler and Richards acknowledged the continued lack of high-quality studies and underscored that most studies are conducted with psychotherapy trainees and that the effects of supervision on more senior therapists are unclear. They also considered the effects of how supervision is implemented (e.g., theoretical models for supervision, frequency of supervision) but found few studies that evaluated such features. The review methodology and report did not meet recommended standards, such as the PRISMA guidelines, and there are some ambiguity regarding exact inclusion and exclusion criteria. It seems that the review included both quantitative and qualitative studies of both counseling and psychotherapy. Taken together, this wide scope makes it difficult to draw firm conclusions about the effects of supervision in a CBT context that may differ substantially in form and content from other forms of psychotherapy.

In a broad literature review, Watkins [4] conducted a semi-systematic review mainly based on previous systematic reviews. The review focused on the effects of psychotherapy supervision on patient outcomes and found three experimental studies of adequate scientific quality but only one of these three studies concerned a structured treatment within the broader framework of CBT (Problem Solving Treatment). The review by Watkins is more qualitative than quantitative, and no general conclusions can be drawn regarding the effects of supervision. However, the results from the three included studies were generally positive and Watkins’ main conclusion is that empirical evaluation of supervision is clearly possible and that the lack of scientific effort in this area is surprising.

There have been other systematic and semi-systematic reviews on the effects of psychotherapy supervision [e.g., 16, 20, 21], but unfortunately, they share the same methodological limitations as the studies described above. Some general conclusions about the field of supervision can be drawn: it is likely that a systematic literature search will find few empirical studies on the effects on supervision with high scientific rigor [19]. It is also likely that the literature will present with a plethora of supervision theories, models, techniques, and outcome measures. There are few established guidelines for evaluation supervision, and the quality of instruments for measuring supervision effects is uncertain [20]. However, there seems to exist a few supervision studies of high scientific quality and there have recently been a couple of high-quality publications that seems to advance the field further.

To summarize, the value of clinical supervision in psychotherapy is expressed by major educational, governing, and practitioners’ bodies while the evidence base for this practice is unclear. This stands in contrast to the growing demands on evidence-based clinical practice which calls for empirically informed psychotherapy. If it is possible to evaluate the effects of psychotherapy on patients’ well-being and health, the effects of supervision should also be possible to assess empirically, though the mediating mechanisms may be more difficult to uncover [5]. Cognitive-behavioral therapy has gone in the breach for evidence-based practice in psychotherapy, and CBT is now an empirically supported treatment model for a wide range of disorders and problems. However, surprisingly few empirical studies have addressed the topic of clinical supervision that have a fundamental place within psychotherapy training and practice. There are positive examples and an increased interest in this missing piece in psychotherapy education research [21, 22]. Recently, and similar to the trend in psychotherapy research, there has also been an increased awareness of the potential harmful effects of clinical supervision [23]. Unwanted and negative effects of supervision are probably few but there is so far limited data on this important issue [24]. As in psychotherapy research, studies on the effects of clinical supervision should arguably be designed to be able to identify and assess any unwanted effects on either supervisees or patients [25]. While there may be a need to further conceptualize the field of clinical supervision in order to gain a better understanding and theoretical model of clinical supervision [26], there is also a need to continuously and systematically review the literature for empirical studies on supervision effects in order to promote an evidence-based practice throughout psychotherapy training and practice. Previous systematic reviews on the effects of clinical supervision have had methodological limitations, have not been rigorously conducted according to established guidelines for systematic reviews, and have included studies of various types and from various theoretical backgrounds, making firm conclusions difficult to draw. There is therefore a need to conduct a systematic review on the effects of clinical supervision that rigorously follows the established guidelines regarding literature search, data synthesis, and reporting.

The goal of the present systematic literature review is to synthesize the effects of clinical psychotherapy supervision on supervisees and their patients in a cognitive-behavioral therapy (CBT) context. The specific study questions are the following:

1. What are the effects of supervision on supervisees’ competence (e.g., skills, behaviors, and attitudes)?
2. What are the effects of supervision on supervisees’ patients’ clinical outcomes, behaviors, and attitudes?
3. What features of supervision are associated with positive and negative outcomes in supervisees and their patients?

## Methods

### Review inclusion criteria

The format, content, and effects of supervision probably vary across contexts, and in order to make a review relevant and meaningful, there must be a balance between too strict and too liberal inclusion criteria. The purpose of a systematic review is to generalize findings across studies and to provide general conclusions, and in order to do so, each inclusion criteria below must be defined based on an assessment of the important processes that make the results reliable and valid for the specified context.

#### Supervisors and supervisees

The population under study in this review consists of supervisors and supervisees. Psychotherapist accreditation varies greatly between countries, and there are no international standards for assessing or labeling different levels of psychotherapy training or competence. However, most countries do have some form of registration/license that authorizes therapists to independently conduct psychotherapy. In this review, supervisors should be psychotherapists that are registered/licensed/accredited or have received similar authorization in their country. Accreditation of psychotherapy supervisors varies even more across countries, and many countries do not have a specific training or education in order to qualify for providing supervision. To allow for this diversity, in this review, supervisors may have any level of supervision competence or training and provide supervision according to any supervision model. This will allow the inclusion of studies on peer-supervision between accredited psychotherapists but not between psychotherapy students.

While most psychotherapy supervision is provided during psychotherapy training, supervision may also be provided to accredited therapists, for example, when learning new methods or treatments. Therefore, the supervisees in this review may be registered/licensed psychotherapists or psychotherapists in training with any level of competence. The supervisees should work in a clinical context and provide ongoing psychotherapy treatment during the supervision period.

For the purpose of this review, counselors, nurses, and physicians with formal psychotherapy education and training who are authorized to provide psychotherapy are included in the term psychotherapists.

#### Supervision

For the purposes of the present review, clinical supervision is defined as a setting in which one psychotherapist instructs and provides training in psychotherapy theories, methods, and skills for psychotherapists who concurrently treat patients. The goal of supervision is to increase the supervisees’ psychotherapy competence and ultimately to improve treatment outcomes and patients’ well-being. The supervision should be focused and tailored for each individual supervisee and his/her professional development. The supervisor should be able to monitor the treatment each supervisee conducts and provide idiosyncratic feedback. The supervision may be conducted in an individual or group setting and in face-to-face meetings or by any other means of communication such as by telephone or video conference.

Broader educational interventions, such as courses or workshops which target psychotherapy skills but are not related to specific ongoing treatments of patients or patient groups, are not considered clinical supervision in this review.

#### Setting

Only supervision in clinical settings may be included, and the psychotherapy provided by supervisees should target psychiatric, psychological, behavioral, emotional, health-related, social issues, or clinical populations.

Supervision cannot be provided free of context, and the theory, form, and content of supervision will to some extent be associated with the theory, form, and content of the therapy provided by supervisees. Supervision that is highly effective in one psychotherapy context may be less relevant in other contexts. In this review, the supervision should provide training in theories, methods, and skills used in the cognitive-behavioral therapy (CBT) framework and the supervisees should also provide treatments within this framework. This includes psychotherapy based mainly on cognitive and behavioral theories and models or more broadly on evolutionary psychology and learning theory. Examples of such psychotherapies are cognitive-behavioral therapy, cognitive therapy, behavioral therapy, dialectical behavioral therapy, meta-cognitive therapy, mindfulness-based cognitive therapy, acceptance and commitment therapy, and problem solving therapy. The common factor in all forms of CBT is the focus on changing behavior in order to change conscious experiences which is expressed in CBT supervision as well.

For the abovementioned reasons, more general forms of psychosocial interventions, such as counseling, that does not use core methods or principles from the cognitive-behavioral therapy framework are not included in this review nor are psychotherapies based mainly on insight or unconscious processes.

#### Types of studies

To be included in this review, studies must be longitudinal and have some form of control condition comparison (e.g., no intervention, waitlist control, treatment as usual or alternative intervention). Experimental and quasi-experimental designs as well as randomized and non-randomized studies may be included.

Uncontrolled studies, case reports, and discursive articles will not be included.

Only studies who evaluate supervision as the main intervention or which present results from a broader intervention in a way so that the effects of supervision are isolated may be included.

Only studies written in English and published after peer-review may be included.

#### Outcome measures

Since there are few standard measures for evaluating psychotherapy supervision, a broad range of outcome measures will be accepted. These should fall within two main categories: (1) competence, e.g., the skills, behaviors, and attitudes of the supervisees/psychotherapists and (2) the clinical outcomes, behaviors, and attitudes of the supervisees/psychotherapists’ patients.

The supervisees’ skills, behaviors, and attitudes must be specified or systematically assessed but may be measured directly or indirectly and may be self-reported or observed. Data from any structured evaluation of the supervisees’ skills, behaviors, and attitudes will be extracted. This includes the use of observational data and objective structured clinical examination as well as standardized instruments such as the Cognitive Therapy Scale. Any reported adverse effects of supervision will also be extracted.

Patients’ clinical outcomes, behaviors, and attitudes may be measured directly or indirectly and may be self-reported or observed. If available, primary outcome variables measured with standardized instruments, such as the Global Assessment of Functioning, the Beck Depression Inventory, Quality of Life Inventory, the Symptoms Checklist 90, or the Client Satisfaction Questionnaire, will be extracted. Any reported adverse effects of supervision reported by patients will also be extracted.

Specific outcome measures are not criteria for eligibility for inclusion in this review.

Secondary outcomes that will be extracted if reported in included studies include any data regarding the supervisors’, the supervisees’, and the patients’ expectations, experience and evaluation of the supervision as well as any feedback data from supervisees’ patients or other benefits or evaluations of the supervision (costs, cost-benefits, feasibility, etcetera).

### Literature search

This review will use a four-step search strategy: (1) Systematic searches for studies will be conducted in the MEDLINE, EMBASE, PsycINFO, and Cochrane Library electronic databases. The key terms for the searches are “psychology,” “supervision,” and “trial,” including any of their synonymous or similar terms (see Additional file 1 for the specific search strategies for each database). (2) Previously published reviews on supervision will be hand searched for studies. (3) All included studies’ reference lists will be hand searched for additional studies. (4) The authors will hand search the reference lists of supervision literature (text books, discursive papers, etcetera) for additional studies.

Trial registers will be searched for any ongoing or unpublished studies. These studies may not be included in the review but may provide valuable background information.

No time restriction for publication will be used in the searches and a complementary search will be conducted just prior to finalizing the manuscript in order to identify any study which is published during the review time.

#### Study screening and selection

First, the titles and abstracts of all identified studies from the literature search will be scrutinized for eligibility and if eligibility is unclear, the full text will be retrieved and scrutinized. The initial screening will be conducted by four of the review authors.

Second, all full texts of studies identified in the initial screening will be retrieved and reviewed by two of the review authors and assessed independently against the inclusion and exclusion criteria. Any disagreements will be resolved by discussion or by a third author if necessary.

### Data extraction

A standardized, piloted form will be used to extract data from the included studies for synthesis and assessment of study quality. Extracted data include study design, study setting; study population; sample size; participant demographics; supervisor competence and characteristics; supervisee competence and characteristics; type of supervision and characteristics; patient group or population; treatment type and characteristics, data collection procedures; outcome measures, quality of outcome measures; main findings and study information for assessment of risk for bias. The supervision of each study will be assessed following the guidelines proposed by Milne et al. [6]. Risk for bias will be assessed using the GRADE checklist [27], the quality of each included study will be assessed with the Jadad checklist [28], and the report of each study will be assessed with the Consort checklist [29]. Two review authors will independently assess the risk of bias, and any disagreements will be resolved by discussion or by a third author if necessary.

Included studies will be entered into systematic review software in order to facilitate review process and author communication. All study data will also be extracted into the software for accessibility.

### Analysis

Characteristics of included studies will be synthesized and presented descriptively according to Data extraction above. Data for each outcome variable will be presented descriptively and if possible the effect sizes (Cohen’s *d*) will be extracted or calculated for each study. We anticipate that outcome variables will vary greatly across studies but if possible, meta-analyses will be considered. If results from included studies cannot be synthesized directly, qualitative summarizations will be conducted in order to provide general conclusions for the study questions.

## Discussion

This systematic review will provide a synthesis on the effects of clinical supervision in a cognitive behavior therapy context and the evidence for using such interventions. This review will include an extensive literature search which will hopefully include all eligible studies in this area. Previous reviews have found few empirical studies on supervision, but we hope that the planned systematic search may identify some additional and more recent studies. In contrast to some of the previous reviews, we have chosen to only include clinical supervision for CBT in this study because we believe that supervision may have different functions and forms and as well as different effects within different theoretical frameworks and therefore may be evaluated separately.

This study may be relevant for a wide audience within psychotherapist training and educational institutions as well as clinical practice. Even though clinical supervision is a fundamental part of professional development very little is known about the effects and beneficial features of clinical supervision [30]. Hopefully, this review may elucidate such questions and lay ground for more empirical studies on clinical supervision.

## Additional file

Additional file 1: Database search macros. (DOCX 23 kb)

## Abbreviations

CBT: Cognitive-behavioral therapy
