# Supplementary material for: The effects of clinical supervision on supervisees and patients in cognitive-behavioral therapy: a study protocol for a systematic review
Source: Syst Rev. 2017 May 11;6:94. doi: 10.1186/s13643-017-0486-7 (PMC5425973; doi:10.1186/s13643-017-0486-7)
Supplement: Additional file 1: — Database search macros. (DOCX 23 kb) [file 13643_2017_486_MOESM1_ESM.docx]

1. MEDLINE (Ovid)

|  | Field labels:   - .ti,ab.=title & abstract - / = MeSH - exp/ = MeSH, exploded - kf = keywords |
| --- | --- |
| 1. exp Psychiatry/ 2. exp Psychology/ 3. exp Psychotherapy/ 4. exp Counseling/ 5. Psychiatric Nursing/ 6. exp Social work/ 7. (therapist* or psychotherapist* or psychologist* or psychiatric nurs* or psychiatrist* or counsel?o?r* or practition* or trainee* or supervisee* or supervisor* or novice* or social work*).ti,ab,kf. 8. ((mental health) adj2 (personnel or staff or employee* or professional* or nurs*)).ti,ab,kf. 9. or/1-8 10. Nursing, Supervisory/ 11. ((clinical or professional or competency based or bug in the eye or bug in the ear or BITE or live or practicum) adj2 supervis*).ti,ab,kf. 12. or/10-11 13. Personal Satisfaction/ 14. exp Patient Satisfaction/ 15. Job Satisfaction/ 16. exp Attitude/ 17. exp Quality Assurance, Health Care/ 18. exp Quality of Health Care/ 19. exp Professional Competence/ 20. exp “Outcome Assessment (Health Care)”/ 21. Program Evaluation/ 22. (quality adj2 (control* or assurance or service* or care or therap*)).ti,ab,kf. 23. (attitude* or satisf* or competen* or abilit* or skill* or outcome* or effects or effectiveness* or evaluation*).ti,ab,kf. 24. or/13-23 25. 9 and 12 and 24 26. limit 25 to english language | |

2. PsycInfo (Ovid)

|  | Field labels:   - .ti,ab.= title & abstract - / = subject headings - exp/ = subject headings, exploded - id = keywords |
| --- | --- |
| 1. exp mental health personnel/ 2. exp counselors/ 3. therapists/ 4. exp psychologists/ 5. exp social workers/ 6. (therapist* or psychotherapist* or psychologist* or psychiatric nurs* or psychiatrist* or counsel?o?r* or practition* or trainee* or supervisee* or supervisor* or novice* or social work*).ti,ab,id. 7. (mental health adj2 (personnel or staff or employee* or professional* or nurs*)).ti,ab,id. 8. or/1-7 9. professional supervision/ 10. practicum supervision/ 11. ((clinical or professional or competency based or bug in the eye or bug in the ear or BITE or live or practicum) adj2 supervis*).ti,ab,id. 12. or/9-11 13. exp ability/ 14. exp satisfaction/ 15. exp attitudes/ 16. exp quality control/ 17. exp "quality of services"/ 18. exp competence/ 19. exp treatment outcomes/ 20. exp evaluation/ 21. exp evaluation criteria/ 22. (quality adj2 (control* or assurance or service* or care or therap*)).ti,ab,id. 23. (attitude* or satisf* or competen* or abilit* or skill* or outcome* or effects or effectiveness* or evaluation*).ti,ab,id. 24. or/13-23 25. 8 and 12 and 24 26. limit 25 to (all journals and english language) | |

3. Embase

|  | Field labels:   - :ab = abstract - :ti = title - exp/ = Emtree term, exploded |
| --- | --- |
| 1. 'mental health care personnel'/exp 2. 'psychologist'/exp 3. 'psychotherapist'/exp 4. 'psychiatrist'/exp 5. 'social worker'/exp 6. therapist*:ab OR psychotherapist*:ab OR psychologist*:ab OR 'psychiatric nurs*':ab OR psychiatrist*:ab OR counsellor*:ab OR counselor*:ab OR practition*:ab OR trainee*:ab OR supervisee*:ab OR supervisor*:ab OR novice*:ab OR 'social work*':ab 7. therapist*:ti OR psychotherapist*:ti OR psychologist*:ti OR 'psychiatric nurs*':ti OR psychiatrist*:ti OR counsellor*:ti OR counselor*:ti OR practition*:ti OR trainee*:ti OR supervisee*:ti OR supervisor*:ti OR novice*:ti OR 'social work*':ti 8. ('mental health' NEAR/2 (personnel OR staff OR employee* OR professional* OR nurs*)):ab 9. ('mental health' NEAR/2 (personnel OR staff OR employee* OR professional* OR nurs*)):ti 10. #1 OR #2 OR #3 OR #4 OR #5 OR #6 OR #7 OR #8 OR #9 11. 'clinical supervision'/exp 12. ((clinical OR professional OR 'competency based' OR 'bug in the eye' OR 'bug in the ear' OR bite OR live or practicum) NEAR/2 supervis*):ab 13. ((clinical OR professional OR 'competency based' OR 'bug in the eye' OR 'bug in the ear' OR bite OR live or practicum) NEAR/2 supervis*):ti 14. #11 OR #12 OR #13 15. 'satisfaction'/exp 16. 'attitude'/exp 17. 'quality control'/exp 18. 'health care quality'/exp 19. 'competence'/exp 20. 'evaluation and follow up'/exp 21. 'outcome of education'/exp 22. (quality NEAR/2 (control* OR assurance OR service* OR care OR therap*)):ab 23. (quality NEAR/2 (control* OR assurance OR service* OR care OR therap*)):ti 24. attitude*:ab OR satisf*:ab OR competen*:ab OR abilit*:ab OR skill*:ab OR outcome*:ab OR effects:ab OR effectiveness*:ab OR evaluation*:ab 25. attitude*:ti OR satisf*:ti OR competen*:ti OR abilit*:ti OR skill*:ti OR outcome*:ti OR effects:ti OR effectiveness*:ti OR evaluation*:ti 26. #15 OR #16 OR #17 OR #18 OR #19 OR #20 OR #21 OR #22 OR #23 OR #24 OR #25 27. #10 AND #14 AND #26 28. #27 AND [english]/lim | |

4. Cochrane Library (Wiley)

|  | Field labels:  ti,ab,kw = Title, Abstract, Keywords |
| --- | --- |
| 1. (therapist* or psychotherapist* or psychologist* or psychiatric nurs* or psychiatrist* or counsel?o?r* or practition* or trainee* or supervisee* or supervisor* or novice* or social work*):ti,ab,kw 2. (mental health NEAR/2 (personnel or staff or employee* or professional* or nurs*)):ti,ab,kw 3. #1 or #2 4. ((clinical or professional or competency based or bug in the eye or bug in the ear or BITE or live or practicum) NEAR/3 supervis*):ti,ab,kw 5. (quality NEAR/2 (control* or assurance or service* or care or therap*)):ti,ab,kw 6. (attitude* or satisf* or competen* or abilit* or skill* or outcome* or effects or effectiveness* or evaluation*):ti,ab,kw 7. #5 or #6      1. #3 AND #4 AND #7 | |
